# Impact of personality functioning and pathological traits on mental wellbeing of older patients with personality disorders

**DOI:** 10.1186/s12888-022-03857-8

**Published:** 2022-03-24

**Authors:** Martine S. Veenstra, Silvia D. M. van Dijk, Renske Bouman, Sebastiaan P. J. Bas van Alphen, Antoinette D. I. Thea van Asselt, Rob H. S. van den Brink, Richard C. Oude Voshaar

**Affiliations:** 1 Department of Psychiatry, University of Groningen, University Medical Center Groningen, Hanzeplein 1, Groningen, the Netherlands; 2 Department of Clinical & Life Span Psychology, Vrije Universiteit Brussel, Brussels, Belgium; 3 Department of Epidemiology and Department of Health Sciences, University of Groningen, University Medical Center Groningen, Groningen, the Netherlands

**Keywords:** Personality disorders, Personality dimensions, Geriatric mental healthcare, Mental wellbeing, Disease burden

## Abstract

**Background:**

Although personality disorders are common and consequential, they are largely ignored in geriatric mental healthcare. We examined the relative contributions of different aspects of personality disorders and comorbid mental disorders to the impairment of mental wellbeing in older adults.

**Methods:**

Baseline data were used of 138 patients who participated in a randomized controlled trial on schema therapy for geriatric mental health outpatients with a full or subthreshold cluster B or C personality disorder. Personality was assessed according to both the categorical and dimensional model of DSM-5. Aspects of mental wellbeing assessed were; psychological distress, positive mental health, subjective health, and life satisfaction. The current study uses baseline data of the RCT to examine the associations between different aspects of personality pathology and mental wellbeing by multivariate regression analysis, controlling for age, sex, level of education, and number of chronic somatic illnesses.

**Results:**

The vast majority of patients (79.0%) had one or more mental disorders in addition to personality disorder. Personality pathology was responsible for the core of the mental health burden experienced by patients, and negated the influence of co-occurring mental disorders when entered subsequently in multivariate analysis. Personality dimensions proved to be highly predictive of mental wellbeing, and this contrasted with absence of influence of personality disorder diagnosis. Although the personality functioning dimensions – and in particular Identity integration (large effect size with partial eta-squared = 0.36) – were the primary predictors of mental wellbeing, personality trait dimensions added significant predictive value to that (Disinhibition 0.25 and Negative affect 0.24).

**Conclusions:**

Personality disorders seriously affect the mental wellbeing of patients, and this overshadows the impact of comorbid mental disorders. In particular personality functioning and pathological traits of the Alternative Model of Personality Disorders (AMPD) of DSM-5 contribute to this impact on mental wellbeing. Alertness for and treatment of personality disorders in geriatric mental healthcare seems warranted.

**Supplementary Information:**

The online version contains supplementary material available at 10.1186/s12888-022-03857-8.

## Background

Comorbidity of mental and personality disorders is common in all age groups. Meta-analyses showed that among patients with a mood disorder, the prevalence of at least one personality disorder ranges from 45% for those with a Bipolar disorder to 60% for those with a Dysthymic disorder [1], and among patients with an anxiety disorder from 35% for those with Post-traumatic stress disorder to 52% for those with Obsessive compulsive disorder [2]. Similar figures are found in studies among older persons referred to specialized mental healthcare. Prevalence of at least one personality disorder ranges from 33 to 58% in these studies [3–5]. In general, about half of the patients in outpatient mental healthcare have a personality disorder [6].

Comorbidity of personality disorders is not inconsequential. A review showed that the presence of a comorbid personality disorder doubled the likelihood of nonresponse to treatment for mood disorders [7]. Similarly, a routine outcome monitoring study in older adults showed that patients with depression and a comorbid personality disorder were more likely to have a poor outcome on functioning than patients without a comorbid personality disorder [8].

Nevertheless, personality disorders are rarely the primary diagnosis for treatment in general or geriatric mental healthcare [8, 9], and structured diagnostic interviews identify significantly more personality disorders than are noted by clinical diagnosis [10, 11]. There may be several reasons for this. Tyrer and colleagues [12], for example, notice that many people with personality disorders do not recognize that they, and not others, are the cause of interpersonal difficulties. In addition, they mention that symptoms of comorbid mental disorders can at times be more prominent than those of personality disorders and can dominate the clinical picture [12]. With respect to geriatric mental healthcare patients, it is further notable that personality disorder phenomenology may change over the lifespan, so that these disorders may have an atypical – and often less pronounced—presentation in older patients [13–16]. Finally, clinicians may be reluctant to diagnose a personality disorder in older patients, out of fear of misinterpreting normal or neurodegenerative changes associated with ageing and the stigma attached to a personality disorder diagnosis [14, 17].

To paraphrase Tyrer and colleagues [12], a reasonable question then for geriatric mental health practitioners to ask is why they should take special notice of personality disorders, when these disorders are difficult to assess and are associated with so many other disorders that seem to be of higher priority to both the patient and therapist? Apart from the above mentioned clinical relevance of personality disorders for the treatment outcome of comorbid mental disorders, the answer lies in the burden of untreated personality disorder for the patient. This burden may be expected to be high, including a lowered quality of life, high levels of psychological distress and a high suicide risk [18–22]. On the other hand, the mental health burden may be primarily determined by comorbid mental disorders instead of the personality disorder. The present study, therefore, examines the relative impact of personality disorders and comorbid mental disorders on mental wellbeing.

The conceptualization of personality pathology has changed in the last decade from a focus on categorical personality disorders to dimensional models. Major examples of dimensional models are the Alternative Model of Personality Disorders (AMPD) of the DSM-5 [23] and the ICD-11 [24]. Both distinguish an overall level of personality functioning (covering self and interpersonal functioning), which indicates the overall severity of personality pathology, and a constellation of personality traits, which describe the stylistic expression of the personality pathology [11]. In this study we will use the AMPD.

The aim of the current study is to examine which aspects of personality pathology are most strongly related to the mental wellbeing of geriatric mental health outpatients with personality disorders. This will clarify the need to address personality disorders, as well as which aspects to address, in treatment of geriatric mental health outpatients with (comorbid) personality disorders. We aim to: (1) examine the relative impact of personality pathology on mental wellbeing, in comparison with the contribution from comorbid mental disorders, (2) test whether the dimensions of the AMPD are more informative on mental wellbeing than the traditional categorical model of personality disorders, and (3) test the assumption that the personality functioning (‘severity’) dimension of the AMPD is more informative than the (‘stylistic’) trait dimensions.

## Methods

### Study design

The present study uses baseline data from an ongoing multi-centre randomized controlled trial [25], regarding the (cost)effectiveness of group schema therapy enriched with psychomotor therapy compared to treatment as usual in specialized mental health care for adults aged 60 years and over with a full or subthreshold cluster B or C personality disorder. Outcome measures were collected at baseline, 6, 12 and 18 months follow up. A total of 145 older adults with a full or subthreshold cluster B or C personality disorder were recruited from eight participating specialized mental health care organizations in the Netherlands. For the current study, we use baseline data of this RCT to study cross-sectional relations between personality pathology and mental wellbeing.

### Study population

Older adults (60 years and older) treated by the participating mental health care centres were screened for eligibility for the study [25]. When the treatment provider suspected a personality disorder, the patient was informed orally and in writing about the study. After informed consent was given by the patients, eligibility was formally evaluated by an appropriately qualified psychologist. This evaluation was standardized and included the Structured Clinical Interview for DSM-5 Personality Disorders (SCID-5-PD) [26]), a checklist with the DSM-5 diagnostic criteria for a number of affective disorders and a cognitive screening with the Montreal Cognitive Assessment [27]. The DSM criterion threshold for diagnosing a personality disorder in older patients has been found to be too strict [13, 28, 29]. For that reason we also included older patients falling short one content criterion for a specific cluster B or C personality disorder, provided that they met the general diagnostic criteria for a personality disorder (denoted here as ‘subthreshold personality disorder’). The specific inclusion criteria for the study were: 1) age of 60 years or older; 2) full or subthreshold cluster B or C personality disorder as confirmed by the SCID-5-PD; 3) mentally able to adhere to the group schema therapy treatment schedule; and 4) giving informed consent after having received oral and written information. Exclusion criteria were: 1) having a bipolar I disorder, psychosis, or substance use disorder needing clinical detoxification, 2) an established neurodegenerative disorder, 3) cognitive impairment defined as a sum score below 23 points on the MoCA, 4) having received schema therapy in the previous year or during the current illness episode, and 5) suicide risk interfering with adequate treatment delivery. All patients who were eligible to participate and signed informed consent were included in the study. Prior to treatment, all participants completed a broad baseline assessment including self-report measures, a structured telephone interview assessing socio-demographic characteristics, psychiatric history, and medical resource use and costs in the past 3 months, and an experience sampling method to assess mood variability by repeated momentary mood assessments on a smartphone. Follow up assessments were conducted at 6, 12 and 18 months after baseline. In the present study, we only used baseline data.

### Outcome measures

Mental wellbeing was assessed by the following set of outcome measures: psychological distress, positive mental health, subjective health, and life satisfaction. These measures were assessed at baseline. Together the measures comprise a broad conceptualization of mental wellbeing and the burden caused by mental health problems. It covers mental health complaints as well as the experience of good mental health and resilience, and in addition takes into account the impact that mental health problems have on general feeling of health and satisfaction with life as a whole.

#### Psychological distress

Psychological distress was assessed by the Brief Symptom Inventory-53 item version (BSI-53), which is a shorter version of the Symptom Checklist-90 (SCL-90) [30]. We used the BSI-53 sum score, which reflects the overall level of psychological distress experienced by the person over the last week, with higher scores indicating more distress. The BSI is well validated for older adults and is preferred in this age group because it is shorter than the SCL-90 [31].

#### Positive mental health

Positive mental health was assessed with the Warwick-Edinburgh Mental Well-being Scale (WEMWBS) [32]. This scale focusses on mental health in contrast to mental illness. Mental health and mental illness have been shown to be related but distinct concepts [33], and reduction or absence of mental illness does not necessarily imply good mental health and wellbeing. The WEMWBS consists of fourteen items covering positive affect, satisfying interpersonal relationships and positive functioning. Items are rated on a 5-point Likert scale assessing the frequency of the positive feeling in the past two weeks. A single score is calculated, with higher scores indicating more positive mental health and wellbeing.

#### Subjective health

The Visual Analogue Scale (VAS) of the five level version of the EuroQoL-5D (EQ-5D-5L) was used to assess the respondent’s self-rated health [34]. The EQ-5D-5L is a self-report questionnaire, covering five health dimensions, which is primarily used for economic evaluation of health interventions from a societal perspective. In addition the EQ-5D-5L includes a VAS (EQ VAS), which records the respondent’s self-rated health on a vertical VAS from 0 to 100, where the endpoints are labelled ‘The best health you can imagine’ (100) and ‘The worst health you can imagine’ (0). For the present study, only the VAS score was used.

#### Life satisfaction

Life satisfaction was assessed with Cantril’s ladder [35], which is a single question to rate one’s current life situation on a scale ranging from 0 to 10, with 0 indicating ‘the worst possible life for you’ and 10 ‘the best possible life for you’. Life satisfaction is a conceptualization of subjective wellbeing which stresses the cognitive evaluation of respondents of their life situation, in contrast to their emotional evaluation, which covers feelings such as happiness.

### Determinants

To examine which aspects of personality pathology are most strongly related with mental wellbeing in older patients with personality disorders, and to compare this with the impact of other mental disorders, the following determinants were studied:

#### Personality functioning

The Severity Indices of Personality Functioning – Short Form (SIPP-SF) [36] assesses five core domains of (mal)adaptive personality functioning defined in the DSM-5 alternative dimensional personality disorders model, namely: Identity integration, Self-control, Relational functioning, Social concordance, and Responsibility. The 60 items of the questionnaire consist of propositions referring to the last three months, which are answered on four-point Likert scales. The answers range from fully agree to fully disagree, and domain scores are calculated as mean item scores [37], where higher scores imply more adaptive functioning. The construct validity of the SIPP-SF was studied in older adults and the instrument was found to be promising as a measure of impaired personality functioning in older adults [37, 38].

#### Pathological personality traits

Pathological personality traits are the second dimension of the DSM-5 alternative dimensional model for personality disorders (AMPD) and were assessed with the Personality Inventory for DSM-5 – Short Form **(**PID-5-SF) [39]. The 100 items of the PID-5-SF cover the 25 pathological personality trait facets (four items per facet) distinguished in the AMPD. These facets are combined to obtain the five higher-order domain scores (Negative affect, Detachment, Antagonism, Disinhibition, and Psychoticism), used in the current study. The items of the PID-5-SF are rated on four-point Likert scales from 0 (very false or often false) to 3 (very true or often true). Domain scores are computed as average item score, with a higher score indicating a more prominent pathological personality trait.

#### Personality disorders

Personality disorders were assessed with the Structured Clinical Interview for DSM-5 personality disorders (SCID-5-PD), according to the DSM-5 traditional categorical model of personality disorders [26]. The SCID-5-PD was administered by an appropriately qualified psychologist. Cluster B and C personality disorders were assessed, including subthreshold disorders, falling one content criterion short (see ʾStudy populationʾ above). Both individual disorders and number of disorders were studied as determinants of mental wellbeing.

#### Comorbid mental disorders

Comorbid mental disorders were assessed with the aid of a DSM-5 checklist. This checklist summarizes all DSM-5 criteria for the following disorders: Major depressive disorder, Persistent depressive disorder, Generalized anxiety disorder, Panic disorder, Agoraphobia, Social anxiety disorder, Posttraumatic stress disorder, Obsessive compulsive disorder, Somatic symptom disorder, and Illness anxiety disorder. At baseline, the treatment provider checked which criteria were present, taking all available information in the medical records of the patient into account. Both individual disorders and number of disorders were studied as determinants of mental wellbeing.

### Covariates

To control for the influence of patient characteristics, the following characteristics were assessed at baseline: age, sex, level of education, and number of chronic somatic illnesses.

#### Level of education

Level of education was assessed by highest education completed and afterwards categorized into low (up to lower secondary education), medium (vocational education), and high (bachelor degree or higher).

#### Chronic somatic diseases

The presence of chronic somatic diseases was assessed by self-report [40]. We inquired about the presence and current treatment by a physician of, and use of medication for, the following chronic diseases: chronic non-specific lung disease, cardiac diseases, atherosclerotic disease of the abdominal aorta or the arteries of the lower limb, diabetes mellitus, cerebrovascular disease, osteoarthritis, rheumatoid arthritis, malignant neoplasms, high blood pressure, stomach ulcers, bowel disorders, liver disease, epilepsy, allergies, thyroid disease, injuries, serious head trauma, and other chronic diseases. The number of diseases for which the person was currently being treated or using medication for was assessed and used in this study.

### Analyses

Three steps may be distinguished in the analyses, of which the first two were preparatory for the main analyses in step three. First, the demographic and baseline characteristics of the study sample were described by descriptive statistics. Second, it was examined whether the four outcome measures of mental wellbeing indeed assess a common component. This was studied by the inter-correlations between the measures and the extent to which a single component explained the variance of the original measures in principal component analysis. Finally, the associations between different aspects of personality pathology and mental wellbeing were examined by multivariate regression analysis, in which the different measures of mental wellbeing (i.e. psychological distress, positive mental health, subjective health, and life satisfaction) were studied as a multivariate set of interrelated outcomes. Only when a significant association between a determinant and the set of outcome measures was found in this multivariate analysis, were the associations of that determinant with the individual outcome measures tested in separate univariate analyses for each individual outcome measure.

The above multivariate regression analysis was used to address the research questions of this study. It was tested whether (1) personality pathology affects mental wellbeing in addition to comorbid mental disorders, (2) the dimensions of the AMPD are more informative about mental wellbeing than the traditional categorical model of personality disorders, and (3) the personality functioning dimensions of the AMPD are more informative than the personality trait dimensions. This was done by entering blocks of determinants successively (i.e. hierarchically) into the analysis, with stepwise forward inclusion of determinants within the blocks. The blocks of determinants and order of entry were: (preliminary) forced entry of all covariates to control for confounding, (1) comorbid mental disorders (individual disorders and number of disorders), (2) the categorical personality disorders (individual disorders and number of disorders), and (3) the personality functioning and personality trait dimensions of the AMPD. To check the assumption of additivity of the impacts of the different determinants on mental wellbeing, we tested for interaction effects between the number of comorbid mental disorder and personality pathology (both the number of personality disorders and the personality dimensions of the AMPD).

Multivariate regression analysis was performed with the General Linear Model (GLM) Multivariate command of SPSS [41]. Partial eta-squared was used as effect size estimate for the strength of an association between a determinant and outcome. For univariate outcomes this statistic indicates the proportion of variance in the outcome accounted for by the determinant, after the effects of other independent variables in the model were partialled out (i.e. excluded). For multivariate outcomes, the statistic has an equivalent meaning of explained variance, but then in multiple outcomes and taking their covariance into account. A partial eta-squared around 0.06 is considered to indicate a medium size effect and 0.14 or more a large effect [42]. The goodness of fit of the final model was assessed for the univariate outcomes by R-squared, showing the proportion of variance in the univariate outcome accounted for by the set of independent variables included in the model. No comparable statistic is available for multivariate outcomes.

Because we also included subthreshold personality disorders (falling one content criterion short for a full diagnosis), we performed a sensitivity analysis to check the findings of the hierarchical multivariate analysis for personality disorder determinants (both individual disorders and number of disorders) based on criteria for a full diagnosis only.

## Results

### Study sample

Of the 145 participants in the RCT, four had missing data on personality dimensions and three on mental wellbeing measures, which left a sample of 138 participants for the current study.

The demographic and clinical characteristics of the study sample are presented in Table 1. The majority of the participants were female (64.5%), and the age of the participants ranged from 60 to 87 years (mean 68.4; SD 5.1). The educational level was relatively high; 38.4% of the participants had a bachelor degree or higher. The vast majority (79.0%) had one or more comorbid mental disorders which primarily consisted of mood disorders (25.4% Depressive disorder and 37.0% Persistent depressive disorder), and further specific anxiety disorders (13.0% Generalized anxiety disorder and 10.9% Social anxiety disorder), and Post-traumatic stress disorder (16.7%). The majority of participants had one or more full personality disorders (*n* = 92; 66.7%), the remainder (*n* = 46) had one or more subthreshold personality disorders.

**Table 1:** Characteristics of the study sample (*n* = 138)

**Patient characteristics**	
Age in years, mean (SD)	68.4 (5.1)
Female sex, n (%)	89 (64.5)
Educational level	
Low, n (%)	33 (23.9)
Medium, n (%)	52 (37.7)
High, n (%)	53 (38.4)
Number of chronic diseases treated, mean (SD)	1.9 (1.3)
**Comorbid mental disorders**	
Depressive disorder, n (%)	35 (25.4)
Persistent depressive disorder, n (%)	51 (37.0)
Social anxiety disorder, n (%)	15 (10.9)
Panic disorder, n (%)	11 (8.0)
Agoraphobia, n (%)	2 (1.4)
General anxiety disorder, n (%)	18 (13.0)
Obsessive compulsive disorder, n (%)	6 (4.3)
Post-traumatic stress disorder, n (%)	23 (16.7)
Somatic symptom disorder, n (%)	9 (6.5)
Illness anxiety disorder, n (%)	3 (2.2)
None, n (%)	29 (21.0)
Number of comorbid mental disorders, mean (SD)	1.3 (1.0)
**Personality disorders (SCID-5-PD)**	
Avoidant PD	
Fully present, n (%)	49 (35.5)
Subthreshold, n (%)	20 (14.5)
Dependent PD	
Fully present, n (%)	12 (8.7)
Subthreshold, n (%)	8 (5.8)
Obsessive compulsive PD	
Fully present, n (%)	30 (21.7)
Subthreshold, n (%)	21 (15.2)
Histrionic PD	
Fully present, n (%)	1 (0.7)
Subthreshold, n (%)	5 (3.6)
Narcissistic PD	
Fully present, n (%)	4 (2.9)
Subthreshold, n (%)	6 (4.4)
Borderline PD	
Fully present, n (%)	21 (15.2)
Subthreshold, n (%)	19 (13.8)
Antisocial PD	
Fully present, n (%)	1 (0.7)
Subthreshold, n (%)	1 (0.7)
Number of PDs (Full or Subthreshold), mean (SD)	1.4 (0.7)
**Personality functioning (SIPP-SF)**	
Self-control, mean (SD)	3.0 (0.6)
Identity integration, mean (SD)	2.4 (0.6)
Responsibility, mean (SD)	3.3 (0.5)
Relational capacities, mean (SD)	2.6 (0.6)
Social concordance, mean (SD)	3.1 (0.5)
**Personality traits (PID-5-SF)**	
Negative affect, mean (SD)	1.4 (0.6)
Detachment, mean (SD)	1.1 (0.6)
Antagonism, mean (SD)	0.3 (0.4)
Disinhibition, mean (SD)	0.9 (0.5)
Psychoticism, mean (SD)	0.5 (0.5)

### Mental wellbeing

Principal component analysis showed that by default a single component would be extracted, which explained 64.4% of the variance in the four outcome measures and correlated strongly (0.79 or more) with the individual measures. The scree plot of the analysis, however, showed that a two components solution would also be possible. In this solution the second component explained another 17.3% of the variance. After oblique rotation of the two components with Oblimin, one of the components mainly related to the two mental health outcomes (Psychological distress and Positive mental health) and the other to the more general outcomes (Subjective health and Life satisfaction). The two components were, however, strongly correlated (0.57), which confirmed that a single component solution was most indicated. Therefore, analysis of the four mental wellbeing outcome measures should be performed by multivariate analysis techniques in which the outcomes are considered as clearly related. If a significant multivariate effect is found, the subsequent univariate analyses may however be expected to show some differences between results for the two mental health outcomes on the one hand and for the two more general outcomes on the other.

### Determinants of wellbeing

Table 2 shows the uncontrolled associations of individual determinants with the multivariate outcome measure, and if significant, also with the univariate outcome measures. Eta-squared instead of partial eta-squared statistics are presented as effect size estimates, because there are no other independent variables to partial out in the bivariate associations studied here.

**Table 2:** Bivariate associations between predictors and multivariate outcome

*Predictor*	Multivariate outcome			Univariate outcomes											
	***Mental wellbeing***			***Psychological distress***			***Positive mental health***			***Subjective health***			***Satisfaction with life***		
	**Pillai’s Trace** **eta**^**2**^	**F**	**p**	**eta**^**2**^	**F**	**p**	**eta**^**2**^	**F**	**p**	**eta**^**2**^	**F**	**p**	**eta**^**2**^	**F**	**p**
***Patient characteristics***															
Age	.04	1.32	.265												
Female sex	.01	0.49	.744												
Educational level	.04^a^	1.24	.275												
Number of chronic diseases	.09	3.12	.017	.04	5.45	.021	.03	3.79	.054	.08	11.66	.001	.04	6.26	.014
***Comorbid mental disorders***															
Depressive disorder	.02	0.49	.743												
Persistent DD	.05	1.74	.145												
Social anxiety disorder	.02	0.54	.706												
Panic disorder	.04	1.43	.228						.						
Agoraphobia	.02	0.80	.528												
Generalized anxiety disorder	.01	0.31	.868												
OCD	.06	2.15	.078												
PTSD	.08	2.74	.032	.08	11.04	.001	.04	5.32	.023	.02	2.38	.126	.02	2.71	.102
Somatic symptom disorder	.01	0.18	.947												
Illness anxiety disorder	.04	1.48	.211												
Number of comorbid mental disorders	.12	4.28	.003	.11	16.87	.000	.06	8.88	.003	.02	2.76	.099	.02	3.12	.080
***Personality disorders***															
Avoidant PD	.03	1.04	.390												
Dependent PD	.02	0.72	.583												
Obsessive Compulsive PD	.03	1.15	.335												
Histrionic PD	.04	1.18	.322												
Narcissistic PD	.06	2.16	.077												
Borderline PD	.04	1.52	.200												
Antisocial PD	.03	1.06	.377												
Number of PDs	.02	0.70	.595												
***Personality functioning***															
Self-control	.31	14.46	.000	.26	47.43	.000	.12	18.71	.000	.02	2.96	.088	.01	0.67	.417
Identity integration	.50	31.60	.000	.37	76.53	.000	.43	98.69	.000	.12	17.94	.000	.14	21.65	.000
Responsibility	.18	7.36	.000	.14	22.60	.000	.15	23.24	.000	.03	4.20	.042	.04	5.73	.018
Relational capacities	.24	10.48	.000	.18	28.59	.000	.21	36.22	.000	.06	8.13	.005	.05	7.49	.007
Social concordance	.21	8.55	.000	.18	28.07	.000	.08	11.08	.001	.02	2.11	.149	.00	0.26	.613
***Personality traits***															
Negative affect	.37	18.99	.000	.34	68.87	.000	.20	32.31	.000	.16	24.61	.000	.17	26.87	.000
Detachment	.39	20.58	.000	.29	55.52	.000	.32	62.43	.000	.11	15.65	.000	.14	22.33	.000
Antagonism	.09	3.34	.012	.05	6.78	.010	.02	2.64	.107	.00	0.09	.764	.00	0.59	.442
Disinhibition	.33	16.16	.000	.27	49.65	.000	.15	23.59	.000	.02	3.17	.077	.01	0.77	.383
Psychoticism	.25	10.52	.000	.22	38.45	.000	.09	13.71	.000	.02	2.16	.144	.02	2.03	.156

^a^Pillai’s trace = .072 and eta squared = .036

Significant associations with the multivariate outcome of mental wellbeing were found for: Number of chronic diseases, Post-traumatic stress disorder, Number of comorbid mental disorders, and all dimensions of Personality functioning and Pathological traits. None of the categorical personality disorder diagnoses, nor the number of personality disorder diagnoses, were associated with mental wellbeing. Medium effect sizes were found for Number of chronic diseases, Post-traumatic stress disorder, Number of comorbid mental disorders, and the Pathological trait Antagonism (eta-squared 0.08 to 0.12) and large effect sizes (eta-squared 0.18 to 0.50) for all other dimensions of Personality functioning and Pathological traits. For all these determinants except Number of chronic diseases, the associations with the two mental health outcomes were somewhat stronger than with the two more general outcome measures (eta-squared 0.05 to 0.37 for Psychological distress and 0.02 to 0.43 for Positive mental health versus 0.0 to 0.16 for Subjective health and 0.0 to 0.17 for Life satisfaction).

Determinants found to be predictive in the hierarchical multivariate prediction analysis of mental wellbeing, are shown in Table 3. Of the block of comorbid mental disorder determinants, entered first to the model already including the control variables, only the number of disorders proved to be a significant determinant of mental wellbeing, with a partial eta-squared of 0.12. Of the personality disorders entered subsequently, none of the disorders nor the number of disorders added significantly to the prediction of mental wellbeing. From the block of personality dimensions considered last, the personality functioning dimension Identity integration was added first to the model, followed by the personality pathology dimensions Disinhibition and Negative affect. In the final model, the partial eta-squared for the number of comorbid mental disorders had dropped to 0.01 and was no longer significant, while the partial eta-squared for Identity integration was 0.36, for Disinhibition 0.25, and for Negative affect 0.24. In particular, a higher score on Identity integration predicted more positive mental health (partial eta-squared 0.31), but also less psychological distress (0.18), better subjective health (0.05) and more satisfaction with life (0.08). For Disinhibition and Negative affect, a higher score was associated with more psychological distress (partial eta-squared 0.18 for Disinhibition and 0.20 for Negative affect). A higher score on Negative affect in addition predicted less positive mental health (0.04), poorer subjective health (0.10) and less satisfaction with life (0.11). The goodness-of-fit of the final model (not shown in the table) was better for the mental health outcomes Psychological distress (R-squared = 0.66) and Positive mental health (0.57) than for the more general outcomes Subjective health (0.31) and Life satisfaction (0.29). No significant interaction effects were found between the number of comorbid mental disorders and personality pathology (i.e. number of personality disorders and personality dimensions of the AMPD); all *p* > 0.10.

**Table 3:** Multivariate predictors of mental wellbeing

Predictor^a^	Multivariate outcome			Univariate outcomes																			
	*Mental wellbeing*			*Psychological distress*					*Positive mental health*					*Subjective health*					*Satisfaction with life*				
	Pillai’s Trace Partial eta^2^	F	p	B	SE(B)	F	p	Partial eta^2^	B	SE(B)	F	p	Partial eta^2^	B	SE(B)	F	p	Partial eta^2^	B	SE(B)	F	p	Partial eta^2^
***Model 1: Blocks 1 (Comorbid mental disorders) and 2 (Personality disorders)***																							
Number of comorbid mental disorders	.12	4.35	.002	0.21	0.05	16.50	.000	.11	-2.04	0.65	9.74	.002	.07	-2.19	1.43	2.33	.129	.02	-0.23	0.15	2.40	.124	.02
***Model 2: Blocks 1 to 3 (Personality dimensions)***																							
Number of comorbid mental disorders	.01	0.35	.844	0.04	0.04	1.29	.259	.01	-0.20	0.54	0.14	.710	.00	-0.17	1.45	0.01	.906	.00	-0.00	0.14	0.00	.988	.00
Identity integration	.36	16.00	.000	-0.33	0.07	25.24	.000	.18	7.06	0.98	51.99	.000	.31	6.30	2.62	5.77	.018	.05	0.86	0.26	10.80	.001	.08
Disinhibition	.25	9.80	.000	0.36	0.07	25.86	.000	.18	-1.85	1.06	3.05	.084	.03	2.19	2.84	0.60	.441	.01	0.53	0.28	3.55	.062	.03
Negative affect	.24	8.97	.000	0.34	0.06	29.00	.000	.20	-2.18	0.95	5.28	.023	.04	-8.98	2.54	12.49	.001	.10	-0.96	0.25	14.29	.000	.11

^a^Predictors in order of entry into the model. All analyses controlled for age, gender, educational level, and number of chronic somatic diseases currently being treated or using medication for

When only fully present personality disorders were considered in the sensitivity analysis (See table in Additional File 1), the number of personality disorders was found to add to the predictive power of number of comorbid mental disorders, and the presence of an Avoidant personality disorder also proved to add to that marginally (*p* = 0.04). The number of personality disorders showed a partial eta-squared of 0.11 with mental wellbeing and an Avoidant personality disorder of 0.08. Number of personality disorders remained significant (with partial eta-squared of 0.09) after inclusion of the personality dimensions (which again consisted of Identity integration with eta-squared 0.31, Disinhibition 0.24, and Negative affect 0.22), but an Avoidant personality disorder was no longer significant (*p* = 0.19). Number of personality disorders was in particular related to worse subjective health (*p* = 0.02), with a partial eta-squared of 0.05.

## Discussion

This study examined the impact of personality pathology on mental wellbeing of geriatric psychiatric outpatients with a full or subthreshold cluster B or C personality disorder. The study shows that in these patients mental wellbeing is primarily determined by the personality functioning and personality trait dimensions of the AMPD. These dimensions (with the exception maybe of the personality trait Antagonism) are strongly related to mental wellbeing, showing large effect sizes both before and after correction for confounders and psychiatric diagnoses (i.e. comorbid mental disorders as well as categorical personality diagnoses). Within these dimensions, the personality functioning domain of Identity integration proves to be of primary importance for mental wellbeing, but closely followed by the personality traits Disinhibition and Negative affect, which show substantial additional predictive value for the level of mental wellbeing of patients. The other personality functioning and personality trait dimensions (with the exception of Antagonism) are strongly related to mental wellbeing, but their predictive value for mental wellbeing proves – at least in this study – to overlap with the three primary dimensions mentioned above.

Regarding our specific research questions, we first saw that personality pathology is responsible for the core of mental health burden experienced by the patients, of whom the vast majority (79.0%) also suffered from other mental disorders. Once confounding factors were controlled for, only the Number of comorbid mental disorders contributed to the prediction of mental wellbeing, and this predictive power was lost when personality pathology was added to the prediction. Hence, our question regarding the need for geriatric mental health practitioners to take heed of personality pathology in addition to comorbid mental disorders, may be answered confirmatively with great conviction. Personality pathology seriously affects mental wellbeing and overshadows the impact of comorbid mental disorders, if present. This adds to the established negative influence of personality pathology on the effectiveness of treatment for comorbid mental disorders, noted in the Introduction [7, 8]. Therefore, also in geriatric mental health care, presence of personality pathology should be checked and scrutinized if present.

Second, we found that in our study sample of patients who all had a full or subthreshold personality disorder, the personality dimensions of the AMPD are highly predictive of mental wellbeing. This contrasted with the absence of any influence of personality disorder diagnosis on mental wellbeing. Of course, the latter may be partly due to restriction of range, as all participants had a personality disorder, and patients with a specific personality disorder diagnosis were therefore compared to patients with at least one other (full or subthreshold) personality disorder. However, the number of personality disorders diagnosed for the person was not found to be predictive of mental wellbeing either. Therefore, it may be concluded that within patients with serious personality pathology, the personality dimensions of the AMPD are much more informative about mental health burden than the traditional categorical model of personality disorder diagnosis (or – as seen above – comorbid mental disorder diagnosis). This underscores the validity of the AMPD model of personality pathology but is also of clinical relevance, as personality dimension scores may facilitate the targeting of personality treatment to the individual patient needs [43, 44]. Hopwood [43], for example, suggests that level of personality dysfunction may be used to indicate level of risk (e.g. for self-harm or treatment disengagement), prognosis, and intensity of treatment required, and may in addition be used as a common outcome measure to monitor treatment progress. Common factors identified in psychotherapy research, such as empathy, therapeutic alliance and expectancy effects, are recommended for targeting personality dysfunction [43]. Pathological traits, on the other hand, specify the patient-specific personality problems and should be addresses by targeted, theory-specific techniques, after the hierarchically organized traits have been disentangled to identify the specific trait facets underlying the maladaptive trait domain scores [43].

Finally, although the personality functioning dimensions – and in particular Identity integration – are the primary predictors of mental wellbeing, personality trait dimensions proved to provide additional – i.e. unique—predictive value too. This indicates that personality functioning is a good indicator of personality pathology severity (when operationalized as impact on mental wellbeing) but that pathological personality traits add to that. Such incremental validity of personality traits to personality functioning was also found in studies which operationalized severity of personality pathology by impact on psychosocial functioning instead of mental wellbeing [45, 46], or on psychological distress specifically [47]. In our study, all traits except Antagonism were strongly related to mental wellbeing. Disinhibition and Negative affect were the traits which were entered successively to Identity integration in the prediction model, but this may be a chance finding, since other traits showed comparable bivariate associations with mental wellbeing (see Table 2). Our results show that the notion in the AMPD that the personality functioning dimensions capture the overall severity of personality pathology and the personality trait dimensions the stylistic expression of the personality pathology (see for example [11, 43]), is too simple. Both groups of dimensions independently contribute to the impact of personality pathology on mental wellbeing, and hence to personality pathology severity. The AMPD was developed on the notion that all personality disorders share core elements of maladaptive personality functioning, in addition to some specific pathological personality traits. These core elements of personality functioning refer to adaptive capacities, which are expected to develop over the person’s life course, and include the capacities to exert control over impulses and emotions, to respect and value oneself and others, and to develop and maintain intimate relationships [36]. Insufficient development of these capacities is expected to capture the level of maladaptive functioning due to personality pathology, and personality traits are not expected to add to that level. It may, therefore, be important to distinguish between ‘level’ and ‘severity’ of personality pathology. Personality functioning may determine the level of personality pathology but not its severity in terms of mental health burden. Different types of personality pathology may share the same level of personality pathology, but may differ in how burdensome they are for the individual. In this study, Antagonism, for example, was not found to be appreciably related to mental wellbeing, in contrast to the other personality trait dimensions of the AMPD. Both from a theoretical and clinical point of view, it may be of considerable interest to study differences in how people experience different types of personality pathology.

Regarding the individual mental wellbeing outcomes, psychopathology – and in particular the personality dimensions – were found to be more closely related to the mental health outcomes (Psychological distress and Positive mental health) than to the more general outcomes (Subjective health and Life satisfaction). The principal predictor of mental wellbeing, Identity integration, for example, showed a bivariate association with Psychological distress with an eta-squared of 0.37 (which corresponds with a correlation r of 0.61) and of 0.43 (*r* = 0.66) with Positive mental health, compared to 0.12 (*r* = 0.35) with Subjective health and 0.14 (*r* = 0.37) with Life satisfaction. One exception may be the personality trait dimension of Negative affect, which showed a less clear difference between its bivariate associations with the mental health outcomes (Psychological distress eta-squared of 0.34; *r* = 0.58 and Positive mental health 0.20; *r* = 0.45) compared to the more general outcomes (Subjective health 0.16; *r* = 0.40 and Life satisfaction 0.17; *r* = 0.41). These latter, relatively strong, associations with the more general outcomes may be the reason why Negative affect was one of the two personality trait dimensions which proved to have additional predictive value for mental wellbeing next to the personality functioning dimension of Identity integration. This may also be an important element to understand why personality trait dimensions prove to assess more than the stylistic expression of the personality pathology and also contribute independently to the level of personality pathology severity. Taken together, however, the psychopathological predictors studied here predicted the mental health outcomes better than the more general outcomes, with proportions of variance explained (R-squared) by the combined predictors of 0.66 for Psychological distress and 0.57 for Positive mental health, compared to 0.31 for Subjective health and 0.29 for Life satisfaction. This corroborates our expectation from the principal component analysis that somewhat different results could be expected for the mental health outcomes compared to the more general outcomes, due to the possible two components solution distinguishing these two groups of mental wellbeing outcomes.

Identity integration was found to be the principal determinant of the impact of personality pathology on mental wellbeing. This personality functioning dimension refers to “the capacity to see oneself and one’s own life as stable, integrated, and purposive” [48], and addresses impairments in self functioning, which together with impairments in interpersonal functioning are thought to constitute problems in personality functioning common to all personality disorders. Our study underscores the importance of adequate concepts of self as stable and worthwhile, and one’s life as purposeful for mental wellbeing. Identity integration explained half of the variance in mental wellbeing in bivariate analysis (eta-squared = 0.50) and more than one third (partial eta-squared = 0.36) of the variance not explained by the other predictors in the multivariate prediction model.

Disinhibition and Negative affect proved to add predictive value for mental wellbeing. These personality trait dimensions refer to an “Orientation toward immediate gratification, leading to impulsive behaviour driven by current thoughts, feelings and external stimuli, without regard for past learning or consideration of future consequences” and “Frequent and intense experiences of high levels of a wide range of negative emotions (e.g., anxiety, depression, guilt/shame, worry, anger) and their behavioural (e.g., self-harm) and interpersonal (e.g., dependency) manifestations”, respectively [23]. These traits may seriously affect mental wellbeing and explained about a quarter (partial eta-squared = 0.25 and 0.24, respectively) of the variance in mental wellbeing not explained by other predictors in the multivariate prediction model. Negative affect specifically proved to be the personality characteristic most closely related to the more general mental wellbeing outcomes of Subjective health and Life satisfaction, both in the bivariate and multivariate analyses, showing correlations with these outcomes of 0.40 and 0.41, respectively.

For all determinants studied we assumed that their effects on mental wellbeing were additive, i.e. independent of each other. We checked this for the main determinants of this study, i.e. number of comorbid mental disorders and personality pathology (both number of personality disorders and personality dimensions of the AMPD). No significant interactions were found. We therefore conclude that there is no indication that the impact of comorbid mental disorders on mental wellbeing is dependent on the extent of personality pathology, nor the other way around.

### Study strengths and limitations

This is the first study on the impact of personality pathology on mental wellbeing in a large sample of geriatric mental health outpatients. It addressed the impact of personality disorders, as well as personality functioning and personality trait dimensions.

The study’s main limitation is its restriction to patients with a confirmed (full or subthreshold) personality disorder, who in addition agreed to participate in a randomized controlled trial on treatment for their personality disorder. Therefore, all included patients will have experienced at least a minimum level of disease burden from their personality pathology, and this may have affected the relative impact of personality pathology and comorbid mental disorders found. In addition, we excluded patients with a comorbid severe current mental illness, an established neurodegenerative disorder, cognitive impairment, or high suicide risk. This too will have reduced the relative impact of comorbid mental disorders to that of personality pathology on mental wellbeing. However, the vast majority of patients studied (79.0%) did experienced comorbid mental disorders, and the epidemiological studies reviewed in the Introduction showed that up to half of geriatric mental outpatients may be expected to have personality disorders, albeit often undetected and untreated. Comorbidity of mental disorders and personality disorders may therefore be expected to be the rule rather than the exception. Future studies should, however, clarify the generalizability of our study findings to geriatric mental health outpatients more broadly.

The inclusion criteria for the study in addition led to a restriction in range of personality pathology studied. Only patients with at least one full or subthreshold personality disorder, and thus a minimum level of maladaptive personality functioning and traits, were included. This may have reduced the strength of the associations between personality pathology and mental wellbeing, as persons without the specific personality disorder or dimension examined will always have had personality pathology on another dimension or disorder. The comparison group never included persons without personality pathology, which reduces the strength of associations of personality pathology with other variables. Nonetheless, strong associations were found between personality dimensions and mental wellbeing. This underscores the potency of these associations.

The associations of comorbid mental disorders with mental wellbeing may have been underestimated because we did not assess the severity of these individual disorders, but only their presence and number. This may have resulted in an underestimation of the disease burden of these comorbid mental disorders and hence of their impact on mental wellbeing.

Another limitation is that cross-sectional studies can’t establish causal pathways. An association between personality pathology and mental wellbeing does not necessarily mean that personality pathology impairs mental wellbeing, or the other way around. It only means that both are related. The terms ‘determinant’ and ‘impact’, as employed in this text, are used in the statistical sense, as indicating the presence and strength of an association.

Finally, we deliberately deviated from DSM-5 criteria for a personality disorder, and also included patients with a subthreshold personality disorder (falling one content criterion short, provided that they met the general criteria for a personality disorder) in the randomized controlled trial on schema therapy and the current study. This may have affected the associations of personality disorders with mental wellbeing. Our sensitivity analysis showed that – contrary to the main analysis which also included patients with a subthreshold personality disorder – number of full personality disorders added predictive value for mental wellbeing to comorbid mental disorders, which remained significant after inclusion of personality dimension in the prediction model. This indeed suggests that the association between personality disorders and mental wellbeing was reduced by including patients with a subthreshold disorder in the group with, compared to including them in the group without, a ‘personality disorder’. Our considerations to include subthreshold personality based on empirical evidence in older patients [13, 28, 29], may therefore be questioned and our findings underscore the need to study the adequacy of personality disorder criteria in older patients further. Inclusion of patients with a subthreshold personality disorder did, however, not affect the main findings of our study. The impact on mental wellbeing of personality dimensions was considerably larger than of personality disorders.

## Conclusions

Our study showed that personality pathology may seriously affect mental wellbeing of geriatric mental health outpatients, in addition to any comorbid mental disorders. Personality disorder diagnoses do not constitute good indicators of this mental health burden, in contrast to the personality dimensions of the AMPD. Assessing severity of personality pathology merely by personality functioning dimensions, as suggested in the AMPD, would however be incorrect. Pathological personality traits may add significant mental health burden to the personality functioning dimensions.

Personality pathology should be seriously addressed in geriatric mental healthcare. The prevalence of personality disorders is known to be high in this setting, and the current study shows that personality pathology may constitute a great burden on mental wellbeing. Alertness for and treatment of personality disorders in geriatric mental healthcare seems warranted.

## Supplementary Information

**Additional file 1: Table. **Sensitivity analysis considering full personality disorders only: multivariate predictors of mental wellbeing.

## Data Availability

The datasets used and/or analysed during the current study are available from the corresponding author on reasonable request.
