# Supplementary material for: Impact of personality functioning and pathological traits on mental wellbeing of older patients with personality disorders
Source: BMC Psychiatry. 2022 Mar 24;22:214. doi: 10.1186/s12888-022-03857-8 (PMC8944148; doi:10.1186/s12888-022-03857-8)
Supplement: Supplementary file 1 — Additional file 1: Table. Sensitivity analysis considering full personality disorders only: multivariate predictors of mental wellbeing. [file 12888_2022_3857_MOESM1_ESM.pdf]

Supplement: Table Sensitivity analysis considering full personality disorders only: multivariate predictors of mental wellbeing

Predictor[#]	Multivariate outcome *Mental wellbeing*			Univariate outcomes																			
				Psychological distress					*Positive mental health*					*Subjective health*					*Satisfaction with life*				
	Pillai's Trace Partial eta²	F	p	B	SE(B)	F	p	Partial eta²	B	SE(B)	F	p	Partial eta²	B	SE(B)	F	p	Partial eta²	B	SE(B)	F	p	Partial eta²
Model 1: Block 1 (Comorbid mental disorders)																							
Number of comorbid mental disorders	.12	4.35	.002	0.21	0.05	16.50	.000	.11	-2.04	0.65	9.74	.002	.07	-2.19	1.43	2.33	.129	.02	-0.23	0.15	2.40	.124	.02
Model 2: Blocks 1 and 2 (Personality disorders)																							
Number of comorbid mental disorders	.11	3.74	.007	0.20	0.05	14.70	.000	.10	-1.62	0.65	6.25	.014	.05	-1.75	1.42	1.51	.221	.01	-0.17	0.15	1.28	.261	.01
Number of personality disorders	.11	3.75	.007	0.20	0.08	6.21	.014	.05	-2.52	1.01	6.28	.013	.05	-6.67	2.20	9.16	.003	.07	-.019	0.23	0.69	.409	.01
Avoidant personality disorder	.08	2.55	.043	0.11	0.12	0.80	.372	.01	0.94	1.56	0.36	.547	.00	-3.84	3.41	1.27	.262	.01	0.44	0.36	1.46	.229	.01
Model 3: Blocks 1 to 3 (Personality dimensions)																							
Number of comorbid mental disorders	.01	0.33	.859	0.04	0.04	1,14	.288	.01	-0.13	0.54	0.06	.813	.00	-0.05	1.44	0.00	.975	.00	0.02	0.15	0.01	.919	.00
Number of personality disorders	.09	2.80	.029	0.08	0.05	2.37	.127	.02	-1.41	0.81	3.02	.085	.03	-5.30	2.15	6.08	.015	.05	-0.01	0.22	0.00	.952	.00
Avoidant personality disorder	.05	1.58	.185	0.08	0.09	0.81	.369	.01	-0.75	1.34	0.31	.578	.00	-5.20	3.55	2.15	.145	.02	0.20	0.36	0.52	.473	.00
Identity integration	.31	12.90	.000	-0.33	0.07	21.50	.000	.16	6.91	1.07	41.4	.000	.26	6.64	2.84	5.47	.021	.05	0.76	0.29	6.80	.010	.06
Disinhibition	.24	8.81	.000	0.35	0.07	23.60	.000	.17	-1.84	1.08	2.90	.092	.02	2.70	2.86	0.89	.347	.01	0.48	0.29	2.70	.103	.02
Negative affect	.22	7.98	.000	0.32	0.06	25.48	.000	.18	-1.98	0.96	4.22	.042	.04	-7.96	2.54	9.80	.002	.08	-0.98	0.26	14.36	.000	.11

Predictors in order of entry into the model. All analyses controlled for age, gender, educational level, and number of chronic somatic diseases currently being treated or using medication for.
